# Primary Sjogren’s Syndrome with Bicytopenia: A Case Report

**DOI:** 10.31729/jnma.8406

**Published:** 2024-01-31

**Authors:** Deepankar Raj Pathak, Ashish Kumar Chaurasiya, Rasish Subedi, Prabin Khatri, Pooja Shah

**Affiliations:** 1 Universal College of Medical Sciences, Bhairahawa, Rupandehi, Nepal; 2 Department of Medicine, Universal College of Medical Sciences, Bhairahawa, Rupandehi, Nepal

**Keywords:** *autoimmune diseases*, *case reports*, *cytopenia*, *sjogren's syndrome*

## Abstract

Sjogren's syndrome is a chronic multisystem autoimmune disease presenting a wide variety of clinical manifestations ranging from mild sicca symptoms to severe systemic symptoms involving pulmonary, renal, musculoskeletal, cutaneous, and haematological diseases. These symptomatic diversities can delay the correct diagnosis of Sjogren's Syndrome for a long time. Here, we report the case of a 59-year-old woman with fatigue and weakness in whom bicytopenia was documented. A thorough bicytopenia workup brought us to the final diagnosis of Sjogren's syndrome. Hydroxychloroquine was started which normalized the patient's blood parameters and clinical symptoms. The haematological alterations in Sjogren's syndrome are not pathognomonic. However, only bicytopenia can be the initial presentation of Sjogren's syndrome as in our patient. Therefore, inexplicable bicytopenia in a middle-aged female may alert the clinician about the possibility of Sjogren's syndrome.

## INTRODUCTION

Sjogren's syndrome (SS) is a chronic autoimmune disease, characterized by lymphocytic infiltration and destruction of exocrine glands especially the salivary and lacrimal glands along with occasional extra glandular involvement.^1^ SS occurs spontaneously-primary SS (pSS) or associated with other autoimmune conditions- secondary SS.^2^ Diagnosis depends on the involvement of the exocrine glands and positive; anti- La/SSB and anti-Ro/SSA antibodies.^3^ The prevalence of primary SS is found to be 6.92 per 100,000 person- years.^4^ Primary SS usually presents as 'sicca complex', but may also present with atypical manifestations such as fatigue, arthralgia, and autoimmune cytopenia.^2,5^ This case focuses on a middle-aged woman with pSS, presenting with normocytic normochromic anaemia and leucopenia.

## CASE REPORT

A 59-year-old female presented to our outpatient department in July 2019, with a complaint of fatigue and weakness for the last three months. She did not report fever, rash, abdominal pain, headache, weight loss, and diaphoresis. She had a history of pulmonary tuberculosis with complete remission two years ago. Besides this, she is a known case of bronchial asthma.

On examination, her lower palpebral conjunctiva was pale, but vital signs and the remainder of systemic examinations were unremarkable.

A complete blood count revealed a haemoglobin level of 9.8 g/dL, white blood cell count (WBC) of 2700/mm^3^ and platelet count of 163000/mm^3^. Biochemical analysis showed elevated globulin levels of 5.0 gm/dl and elevated lactate dehydrogenase (LDH) levels of 521 IU/L. The erythrocyte sedimentation rate (ESR) was also significantly raised at 60 mm/hr. Coombs tests both direct and indirect were negative along with normal bilirubin levels and thyroid function. Peripheral blood smear showed normocytic, normochromic red blood cells (RBCs). We initiated a bicytopenia workup after obtaining these results.

Serum protein electrophoresis showed polyclonal hypergammaglobulinemia. HIV, Hepatitis B and C tests were negative. Ultrasound and pap smear were unremarkable. Antinuclear antibody (ANA) showed a homogenous pattern with a high titre (1:320); rheumatoid factor (RF) was positive (28.9 IU/ml). High titer of ANA grabs our attention to probable drug-induced lupus (DIL), as she already had a history of antitubercular drug intake; we performed an extractable nuclear antigens (ENA) panel which indicated strong positivity for SS-A, SS-B, and Ro-52. Anti-dsDNA and anti-histone antibodies were both negative. A bone marrow biopsy was then done which turned out normal, excluding blood malignancies. These results gave a strong impression of SS as the probable diagnosis.

When we repeatedly inquired her about sicca symptoms, she admitted having symptoms of dry eye which were not significant enough to catch her attention. On an ophthalmological examination, Schirmer's test was positive (4 mm right eye, and 5 mm left eye). We also advised her to undergo a labial biopsy, but she denied undergoing an invasive procedure. However, she fulfilled the diagnostic criteria (2016-ACR-EULAR Classification) for pSS.^6^ The classification of SS would apply to any individual who meets inclusion criteria and has a score >4. Our patient had a score of 4, as a result, we diagnosed her with pSS with bicytopenia.

She was treated with hydroxychloroquine 200 mg, twice a day, for three months along with supportive therapy (oral pilocarpine, artificial tear and saliva). After three months, her symptoms had improved drastically with the normalization of all blood tests. As a result, hydroxychloroquine was tapered once daily in the evening. She has been following up for 2 years with complete normalization of her symptoms and blood parameters. She is under a maintenance dose of hydroxychloroquine currently.

## DISCUSSION

Sjogren's syndrome is a multisystem autoimmune disease, associated with B-lymphocyte hyperreactivity with hallmark features of lymphocytic infiltration in salivary and lacrimal glands.^5^ SS can occur spontaneously-primary SS or may be associated with other autoimmune conditions such as rheumatoid arthritis (RA) and systemic lupus erythematosus (SLE).^2^ It is one of the common systemic rheumatic disorders with a prevalence of approximately 0.1% and 4.8%. It predominantly occurs in middle-aged women with a women-to-men ratio of 9:1.3 Its clinical features range from mild sicca symptoms (100%), extreme fatigue (50%), arthralgia to severe systemic symptoms including vasculitis, interstitial lung disease, glomerulonephritis, neuropathy, renal(interstitial nephritis and tubular acidosis) disease and autoimmune cytopenia.^1-3,5^ As well as 4-6% of patients with SS can develop non-Hodgkin lymphoma. Some patients, however, present with negligible or no sicca symptoms at all and may not have complaints regarding the same even if they have such symptoms.^7^

Activated B-lymphocyte specifies the presence of RF, hypergammaglobulinemia and autoantibody to Ro/SSA and La/SSB. These antibodies lead to tissue dysfunction even before inflammation becomes obvious and are associated with earlier disease onset.^1^ Persistently high titre of RF in the absence of either cryoglobulinemia or rheumatic arthritis is suggestive of probable pSS.^5^ In our patient, the RF titre was high with negative anti-CCP and no joint symptoms excluding the possibility of RA. Similarly, skin lesions, peripheral neuropathy and antibodies to HCV were absent ruling out the cryoglobulinemia as well. Thus, we headed towards the probable pSS.

ANA is the essential marker for the detection of autoimmune disorders.^8^ A positive ANA result should be interpreted in relation to other lab investigations besides its staining pattern.^9^ Our patient had positive ANA titre with a homogenous pattern. The common systemic rheumatic diseases associated with homogenous patterns are SLE, MCTD, DIL and juvenile idiopathic arthritis. Therefore, to rule out these autoimmune diseases, we performed an ENA panel which revealed positive anti-SSA/Ro and anti-SSB/La antibodies. Anti-SSA/Ro and anti-SSB/La antibodies are the immune markers used in the detection of Sjogren's syndrome, subacute cutaneous SLE and neonatal lupus syndrome.^8^ However, our patient was not pregnant and had no photosensitive dermatosis pointing us towards probable pSS. Thus, patients with pss can also present with a less common homogenous ANA pattern despite speckled being the more common one, as in our patient.^9^

Anti-Ro/SSA and ESR >50 mm/hr have the strongest prevalence of extra glandular manifestation. Our patient also presented with similar findings. SS diagnosis is based on criteria proposed by ACR-EULAR published in 2016. Hematological abnormalities are a frequent finding in many autoimmune diseases but in SS they account for approximately 25-50%. Anemia occurs in around 34.1% of patients with pSS and most common being anemia of chronic disease with the normocytic normochromic pattern. Our patient also presented with normocytic normochromic anaemia which occurs because of pro-inflammatory cytokines such as overexpression of IL-1, IL-17 and lower expression of IL-4, thus blocking the action or decreasing the production of erythropoietin (EPO).^1,3^

Likewise, leucopenia is seen in 14-42% of pSS which was also seen in our patient. Leucopenia is prompted by the presence of autoantibodies against the cell lineages. Lastly, 5-15% of cases of SS can complicate thrombocytopenia which was not seen in our patient.^3^ Hematological manifestation may not be pathognomonic for pSS; however, bicytopenia can be the sole or initial presentation of pSS as in our patient.

The treatment protocol of pSS is based on the disease process; when extra glandular damage is present, glucocorticoid or immunosuppressive therapy such as TNF inhibitor, hydroxychloroquine, rituximab, methotrexate and cyclophosphamide is used. According to the latest Sjogren's Syndrome Foundation clinical practice guideline, the first line therapy for inflammatory musculoskeletal pain and fatigue associated with pSS is hydroxychloroquine of moderate and weak strength respectively.^10^ Our patient was also prescribed hydroxychloroquine of weak strength (200 mg) which improved her fatigue along with normalization of blood counts.
